# Role of myeloid derived suppressor cells in tuberculosis infection and disease

**DOI:** 10.1186/1471-2334-14-S3-O18

**Published:** 2014-05-27

**Authors:** Nathella Pavan Kumar, Rathinam Sridhar, Vaithilingam V Banurekha, Mohideen S Jawahar, Thomas B Nutman, Subash Babu

**Affiliations:** 1 National Institutes of Health international Center for Excellence in Research, Chennai, India; 2 National Institute for Research in Tuberculosis, Chennai, India; 3 Government Stanley Medical Hospital, Chennai, India; 4 Laboratory of Parasitic Diseases, National Institutes of Allergy and Infectious Diseases, National Institutes of Health, Bethesda, Maryland, USA

## Background

*Mycobacterium tuberculosis* infects 2 billion people worldwide, 90% of infected individuals are able to resist overt disease development and manifest only latent infection. Myeloid derived suppressor cells (MDSCs) are a heterogeneous population of early myeloid progenitors, immature granulocytes, macrophages, and dendritic cells at different stages of differentiation. These cells have the capacity to suppress activities of adaptive immune response mediated by CD4^+^ and CD8^+^ T cells.

## Methods

The frequency of MDSC(CD45+CD14+HLA dR-), Granulocyte derived MDSC (CD45+CD33+HLA dR CD14 CD11b+) and Monocyte derived MDSC(CD45+CD33+HLA dR CD14+CD11b+) was examined by using flow cytometry in pulmonary tuberculosis (PTB)(n=115) and compared with extra-pulmonary TB (ETB)(n=106), latent TB (LTB)(n=102) and healthy controls (HC)(n=73)

## Results

PTB is characterized by significantly elevated frequencies of MDSC (Geometric mean [GM] of 0.5587% in PTB and 0.3095% in EP; *p*=0.0005); G-MDSC (GM of 0.1660% in PTB and 0.3089% in EP; *p*=0.0189) and M-MDSC (GM of 0.1307% in PTB and 0.0756% in EP; *p*=0.0128) when compared with ETB. On the other hand, there was diminished frequency of MDSC (GM of 0.5587% in PTB and 1.064% in LTB; *p*= 0.0060) (GM of 0.5587% in PTB and 1.187% in HC; *p*<0.0001) and G-MDSC (GM of 0.1660% in PTB and 0.4131% in HC; *p*<0.0001) (GM of 0.1660% in PTB and 0.3516% in HC; *p*<0.0001) in PTB group when compared with LTB and HC, respectively.

## Conclusions

The data reveals that MDSC were not only induced during active pulmonary TB infection but also in latent TB and healthy controls after recent exposure to *M. tuberculosis.*

